# An App That Incorporates Gamification, Mini-Games, and Social Connection to Improve Men's Mental Health and Well-Being (MindMax): Participatory Design Process

**DOI:** 10.2196/11068

**Published:** 2018-11-19

**Authors:** Vanessa Wan Sze Cheng, Tracey A Davenport, Daniel Johnson, Kellie Vella, Jo Mitchell, Ian B Hickie

**Affiliations:** 1 Brain and Mind Centre The University of Sydney Sydney Australia; 2 School of Electrical Engineering and Computer Science Science and Engineering Faculty Queensland University of Technology Brisbane Australia; 3 The Mind Room Collingwood Melbourne Australia

**Keywords:** football, mental health, well-being, video games, adolescent, young adult, cell phone, gamification, sport, men’s health, social connection

## Abstract

**Background:**

Men have different mental health needs as compared with women, and women make up the primary audience of most digital mental health interventions. An Australian football-themed (specifically Australian Football League, AFL) app named MindMax incorporating psychoeducation, gamification, mini-games, and social connection was developed in an effort to address this issue.

**Objective:**

The aim of this study was to identify the best way to structure and present MindMax, an app that aims to deliver psychoeducational modules, and create a Web-based community centering on well-being, AFL, and video games for men aged 16 to 35 years who are interested in AFL or video games.

**Methods:**

We conducted 6 participatory design (PD) workshops with people aged 16 to 35 years in 3 cities in Australia, to identify the best way to present MindMax, and contracted a digital development agency to develop MindMax. We then iteratively tested MindMax prototypes with 15 user experience testing interviews across 3 separate time points: 2 before app launch and 1 after app launch.

**Results:**

A total of 40 individuals (25 male and 15 female) participated in the PD workshops, and a total of 15 individuals (10 male and 5 female) participated in user experience interviews. Broadly, participants expressed a preference for activities requiring active engagement that practiced useful skills. They were also sensitive to how content was presented and wanted the ability to customize their own app experience. Although participants agreed that social motivations were important for engagement with an app, they recommended not to mimic existing social networks.

**Conclusions:**

In basing itself strongly within the AFL subculture and by incorporating gamification as well as mini-games, MindMax aimed to tackle mental health help-seeking barriers for people who enjoy AFL or video games, with a particular emphasis on men, and to provide psychoeducation on strategies to increase mental health and well-being. If MindMax is successful, this would indicate that generalizing this approach to other traditional sporting codes and even competitive video gaming leagues (esports) would be fruitful.

## Introduction

### Men’s Well-Being and Internet Interventions

As participants in mental health research are heavily biased toward being female [1,2], research outcomes may not be fully generalizable to men. A growing body of evidence suggests that men’s experiences of mental health problems and treatment differ to those of their female counterparts [3,4]. For example, young men have higher rates of suicide prevalence and lower rates of mental health literacy and health care service access than young women [1,3]. Furthermore, women are more receptive to structured internet health interventions than men [4]. This problem is especially urgent for young people as most mental health problems are developed during young adulthood [5]. Furthermore, mental health outcomes at a young age persist and potentially worsen in the long term, even among those receiving clinical care [6]. A targeted approach to improving mental health that aims to give younger people the tools to manage their own well-being would be helpful in addressing this issue.

Western norms of masculinity (in particular, the emphasis on self-reliance and on silently coping with psychological distress) act as barriers to help seeking [7] and contribute to the worse outcomes displayed by young men. This is exacerbated by the small but significantly higher tendency for young men to avoid addressing their friends’ mental health problems directly and to avoid recommending they seek help from external sources, relative to their female peers [8]. Furthermore, the high levels of mental health stigma that persist in young men and their negative perceptions of mental health professionals work in tandem with the previous to contribute to high reluctance to formally seek help from mental health professionals [7]. Instead, young men tend to seek informal help from the internet, with 1 study on 16- to 24-year-old men finding nearly 55% of their sample reported having done so [7]. The same study further reports that, within their sample, younger men were more likely to seek informal help from the internet than older men.

An approach to internet interventions that enables and informs such informal help seeking would be a natural fit to this pattern of behavior. An evidence-based approach toward men’s mental health and well-being that addresses the problems outlined above should therefore be (1) accessible on the internet, (2) action-based and informal (not clinical), (3) anonymous with the potential for social connection, (4) self-directed, and (5) based in subcultures men are already present in [7]. Importantly, women should not be excluded as in addition to benefiting from the intervention themselves, they can also act as supportive others, connecting these interventions and other mental health initiatives to the men in their lives.

Sports and video games are mainstream topics with significant male fan bases. The sporting code that is the focus of this study, Australian Football League (AFL), is a type of Australian football and enjoys the support of 6 million people across Australia [9]. Similarly, a recent nationwide survey reports that over three-quarters of Australians aged 15 to 34 years play video games and that 70% of all men surveyed were video game players [10]. Both cultures are further combined in esports (competitive video gaming) [11]. esports is a growing industry popular among younger people, with nearly as many millennials preferring watching their favorite esport to watching their favorite traditional sport (40% vs 42%) [12]. Furthermore, many sporting leagues including AFL have partnered with esports teams [11] and video game companies to host esports events [13]. Although they remain distinct subcultures, sports and video games are highly compatible, mainstream in the general population, and well suited to utilization in a mobile health (mHealth) app intended to promote mental health and well-being.

### Gamification and Applied Games

The general usage of games and game features for nonentertainment purposes is known as “applied games” [14]. This includes not just applying commercial video games outside of an entertainment context, for example, psychological therapy [15], but also serious games (video games developed for a primary purpose other than player enjoyment [16]) and gamification [17,18]. In the same way that traditional video game design works to engage both the extrinsic and intrinsic motivation of its players, applied games have inherent “effectiveness potential” [19], where users of interventions that incorporate applied games can be motivated to explore the intervention deeper for additional motivations besides self-improvement. Furthermore, the inherent design characteristics of video games have been shown to be complementary to subjective well-being concepts, for example, the Seligman positive emotions, engagement, relationships, meaning, and achievement (PERMA) model [20,21]. There is also evidence for a positive impact of moderate video game play on well-being [22-25].

Gamification, in particular, has been named a promising strategy with which to promote engagement in digital health interventions [19,26,27]. Although the most well-known gamification elements are points, badges, and leaderboards [28], prioritizing these elements can undermine the complex series of cognitive, emotional, and social affordances that make games intrinsically motivating and enjoyable to play [29]. The most successful mHealth initiatives that incorporate gamification have been carefully designed to include both extrinsic and intrinsic motivators [30,31].

The term *gamification* has been defined as the “use of game design elements in non-game contexts” [17] as well as “a process of enhancing a service with affordances for gameful experiences in order to support user’s overall value creation” [18]. The latter definition by Huotari and Hamari is particularly useful in an mHealth (both mental and physical health) context, with “value creation” potentially being the improvement of the user’s health; the adoption of health behaviors; the provision of a fun, engaging educational experience; or all of the above. This definition also emphasizes the goal of gamification rather than its methods and recognizes that what some individuals may term “game design elements” may not be considered as such by others, complementing the overlap between gamification and other health behavioral change frameworks such as persuasive systems design [32]. Finally, this definition is drawn from a service marketing approach. By viewing the digital health intervention as a core service, it becomes easier to visualize how the components of this service can be enhanced with motivational affordances. Approaching applied games (including gamification) from this perspective could thus lead to a more compatible and natural integration of applied games (including gamification) into mental health care.

Although empirical study of the effects of gamification is still in its infancy, there is evidence, albeit outside of health, that it leads to higher and more involved user engagement with an app or service [33,34]. However, the impacts of gamification within electronic health and mHealth remain poorly understood [35-37]. Although application varies by health domain, many mHealth apps do not utilize gamification [36], and those that do tend to contain limited applications of it [38]. As evidence of individual differences in gamification element preferences is emerging [39], it is clear that to provide the enjoyable and engaging experiences initially hoped for, when gamification (and applied games in general) is applied to mHealth, it must be with due consideration.

### Participatory Design and Knowledge Translation

Although researcher-led mHealth initiatives have a key strength in applying evidence-based best practice, it is often at the expense of user experience. It is difficult to compete with large corporations who invest millions of dollars into creating seamless, intuitive, and engaging user experiences to entertain their consumers. This level of investment is near impossible in academia, which may result in a jarring experience for users accustomed to a contemporary internet experience [40]. Another key tension within mHealth initiatives is that their aims and objectives often act as barriers to uptake, especially among populations that engage the most in behaviors the intervention hopes to reduce (eg, drinking alcohol [41]). It is important to identify how best to present the health and therapeutic content of mHealth initiatives to the target audience. One method of achieving this is through participatory design (PD) [42].

The key principle of PD is to involve all stakeholders of a project in an iterative cycle of design and development [43,44]. This allows them to influence its design to better suit their past, present, and future needs, ideally leading to higher effectiveness and engagement among the target population [1]. Furthermore, when executed well, PD methodologies increase the acceptability of interventions to stakeholders [44] and can be harnessed to make knowledge translation of research outcomes more efficient [43,45]. This is especially important in mHealth, as given the rapid pace of technological development, the field must reduce the lag between health research and translation as much as possible.

### Study Context and Objectives

As part of its daily operations, the Australian Football League Players’ Association (AFLPA) offers mental health and well-being training to more than 800 players across the National League [46]. This training focuses primarily on resilience and well-being. Well-being is a separate construct of positive mental health that is distinct to mental illness [47]. A focus on well-being is more broadly applicable to the general population as both people with and without mental illness can directly benefit from learning how to maintain and improve their well-being. Notably, increasing subjective well-being leads to improvements in individuals’ lives, such as healthier relationships, more positive emotions, increased feelings of autonomy, and increased self-acceptance [21].

In collaboration with Queensland University of Technology and The University of Sydney’s Brain and Mind Centre, the AFLPA obtained funding to execute a multipronged initiative to improve mental health and well-being, focusing on men aged 16 to 35 years but not excluding other groups of people [48]. The app resulting from this collaboration was named MindMax and aimed to deliver a modified version of the AFLPA’s existing mental health programs in a portable, digital format. The target audience is hence men aged 16 to 35 years who are interested in AFL or video games.

MindMax was designed according to the 5 recommendations made by previous research [7] outlined in the first section of the Introduction. For example, educational content was split into multiple small modules lasting around 10 min each, to enable self-directed learning and to give users a choice in what aspects of their well-being they wish to focus on [40]. A secondary aim of MindMax was to create a Web-based community centering on well-being, sports (in this case AFL), and video games. To achieve this aim, the AFLPA engaged a select number of AFL players as ambassadors for the app. Their role would include being spokesperson within the app modules and community area as well as promoting the app to the AFL industry and general public.

Although the basic components of MindMax were drawn from the literature and decided on by researchers and the AFLPA, it was unclear how best to present them in a way that would be acceptable to the target audience. The aim of this study was, therefore, to use PD and user testing methodologies on multiple iterations of MindMax to obtain key insights from end users on how best to present its content, design, and features.

## Methods

### Participant Recruitment

Our recruitment strategies consisted of putting up posters, distributing postcards, and advertising in student mailing lists. We also asked affiliated organizations to assist in recruitment for locations not in Sydney. We reimbursed PD workshop participants with a gift voucher worth Aus $50 and user experience interview participants with a gift voucher worth Aus $30 to thank them for volunteering their time and expertise. The University of Sydney’s Human Research Ethics Committee (Protocol No. 2016/652) approved this project before the start of research activity.

### Phase 1: Participatory Design Workshops

#### Design

The PD methodologies used in this study are based on recommendations by the Young and Well Cooperative Research Centre [44]. Specifically, we adapted the iterative PD and knowledge translation methodology used by Ospina-Pinillos et al [45] to fit the needs of our project.

In phase 1, we held 2 PD workshops in each of 3 Australian capital cities in early September 2016, making 6 workshops in total. The aim of these workshops was to identify how best to frame the well-being concepts discussed in MindMax and, more broadly, how to structure a mental health and well-being app to the intended audience. Moderators took notes during the workshops. Workshops lasted 3 hours and consisted of 3 stages: discovery, evaluation, and prototype.

##### Discovery

Workshop moderators facilitated participant discussion of their knowledge and usage of and preferences for, health and well-being apps/websites. Specifically, participants discussed their preferences for app design and content, their social usage of health and well-being apps, applying gaming concepts to mental health and well-being, and data tracking and privacy. Although moderators focused on mental health in particular, both mental and physical health were discussed.

##### Evaluation

Moderators then presented screenshots of existing health and well-being apps/websites to participants for their critical evaluation. These apps were a combination of popular commercial health and well-being apps (including physical health) as well as output of previous academic and government mental health and well-being mHealth projects. Screenshots portrayed a variety of features of interest, including social connection (dashboard and community pages), gameful elements (mini-games, challenges, and progress bars), and psychoeducation. Marker pens were provided for participants to annotate the screenshots.

##### Prototype

Finally, in the context of the previous 2 stages of discussion, moderators asked participants to design concepts, specifications, or potential user journeys for a mental health and well-being app. Sketchbooks and marker pens were provided for this activity.

### Phase 2: Knowledge Translation

**Figure 1:**
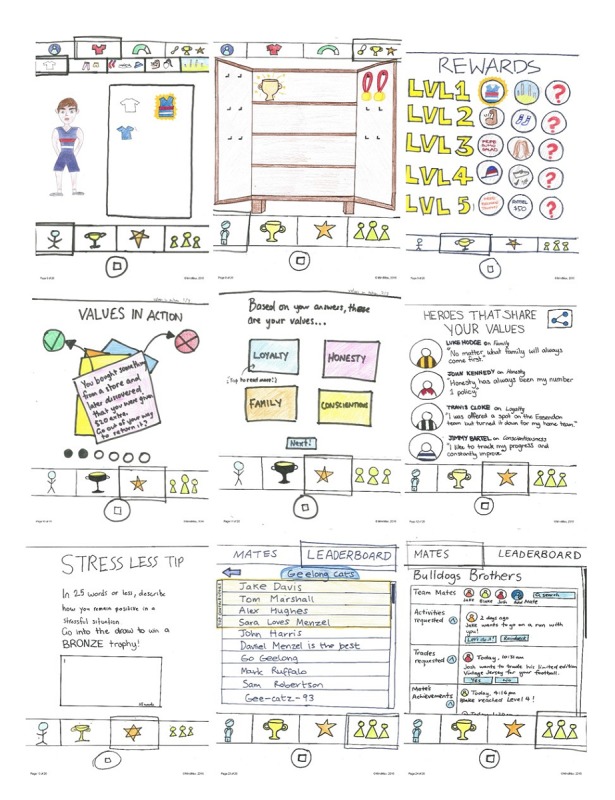
Selected sketches from the knowledge-translated design of MindMax.

Following the PD workshops, all moderator notes and participant artifacts (produced during the evaluation and prototype stages) were collated and analyzed by an independent knowledge translation team consisting of a group of young people (aged under 25 years) who were short-term interns at The University of Sydney’s Brain and Mind Centre. The team adopted an approach similar to descriptive content analysis [49], manually coding the notes and artifacts by 3 overarching semantic themes: content (the information and activities within the app), design (the visual design of the app), and features/concept (the conceptual design and features of the app). The team then used these themes and codes as guidelines to produce a knowledge-translated design of MindMax (see Figure 1).

### Phase 3: User Experience Testing Interviews

#### Timeline and App Details

We presented the outcomes of the PD workshops and the resulting knowledge translation to the AFLPA, who concurrently contracted a digital agency (Long Division Digital, Melbourne) to produce a prototype of MindMax, drawing principles from the outcomes as appropriate and feasible. This prototype and further iterations were tested in one-on-one user experience interviews at The University of Sydney’s Brain and Mind Centre across 3 time points: December 2016, March 2017, and November 2017. MindMax was launched to the public in June 2017. Hence, 2 time points were before launch and 1 time point was after launch. Drawing on previous recommendations in user experience research [50], we aimed for a total of 15 participants (5 participants per round) to allow for as many insights to be captured across multiple iterations of MindMax, as efficiently as possible.

**Figure 2:**
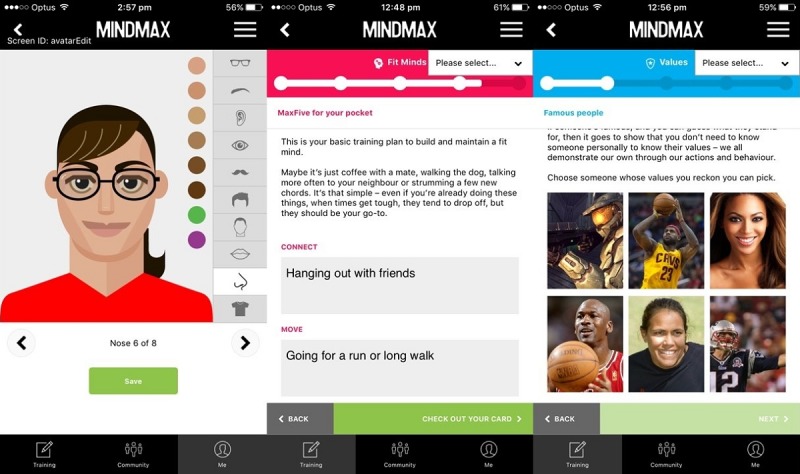
The beta build tested in March 2017. Left: the avatar creation process; middle: a goal-setting activity in Fit Minds; right: an activity in Values.

**Figure 3:**
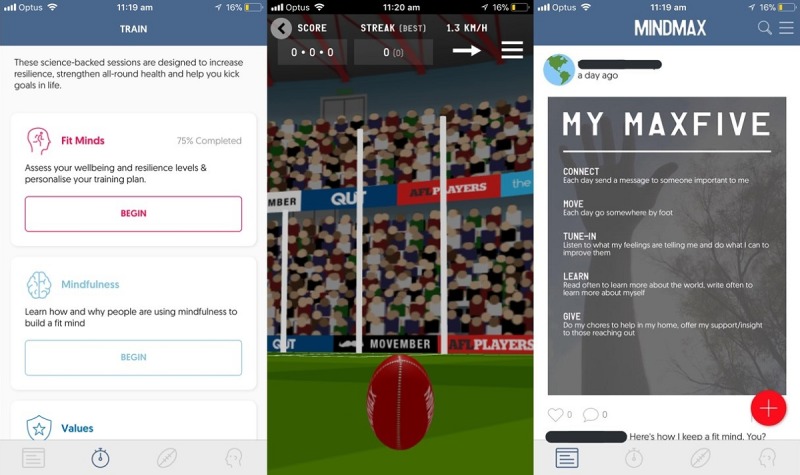
The updated launch version tested in November 2017. Left: the psychoeducational module selection screen following a layout change; middle: the new goal-kicking casual game, “Flick Footy,” which cost “footies” to play. “Footies” are earned by interacting with the social or psychoeducational components; right: an anonymized example of a “shareable” generated by a user after completing Fit Minds.

During the first time point, we tested a hybrid Web-based prototype with limited functionality, and the moderator assisted participants in accessing the app build through a mobile phone internet browser. At this point, only the *Fit Minds* psychoeducational module was available. During the second time point, we tested an in-progress native build with greater functionality (see Figure 2). At this point, 3 psychoeducational modules were available: *Fit Minds*, *Values*, and *Thoughts*. During the third time point, which was 5 months after MindMax was launched on the App Store and Google Play Stores, we tested an updated version of MindMax (see Figure 3) and asked participants to download it onto their mobile phones. At this point, 5 psychoeducational modules were available: *Fit Minds*, *Values*, *Thoughts*, *Mindfulness*, and *Emotions*, as well as “Flick Footy,” a casual game involving kicking a football to score goals.

**Figure 4:**
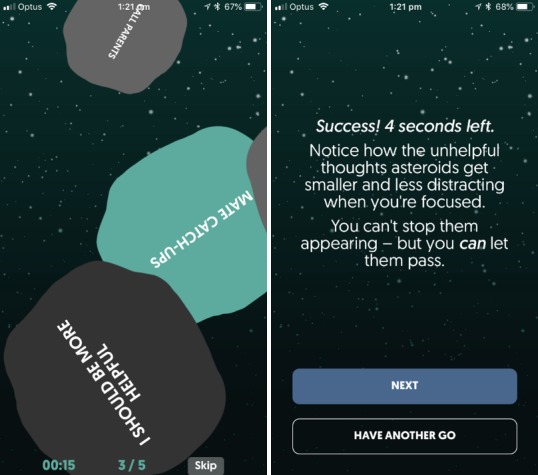
An example of a mini-game used to illustrate the concept of letting unhelpful thoughts pass, in Thoughts.

Along with the social component (community feed), the psychoeducational modules and Flick Footy formed the reward system within MindMax, where completing psychoeducational modules and posting in the community feed earned users points, called “footies,” which could then be spent to play Flick Footy. In addition to this, modules also contained mini-games (see Figure 4) that aimed to allow users to interact with the lessons in a more active way.

Psychoeducation modules were accessible through a tab named “Train/Training” (depending on the version of MindMax) and were designed to last around 10 min. They consisted of information pages, interactive activities, and videos that contained information about the module topic and featured AFL players as spokespersons. The videos with AFL players were a montage of informal interviews relating to the module topic and were presented as a way to get to know another side of the player. During certain points of each module, shareable content (a “shareable”) was generated and posted to the community feed. These posts could be toggled to display to all MindMax users (*public*) or to the user only (*private*).

*Fit Minds* was an introductory module with the objective of creating a “MaxFive” plan to improve well-being, *Values* aimed to help users identify their values and ways to act upon them, *Thoughts* aimed to help users identify and deal with unhelpful thoughts (including a mini-game illustrating the concept of letting unhelpful thoughts pass by; see Figure 4), *Mindfulness* aimed to introduce users to mindfulness meditation, and *Emotions* aimed to help users gain a better understanding of their emotions and how to deal with negative emotions. The *Mindfulness* and *Emotions* modules contained 2 guided audio meditation tracks recorded with 2 different AFL player ambassadors, and users could choose which player to meditate with. Information was presented in a casual, masculine tone to reflect the target audience of the app (men aged 16 to 35 years interested in AFL or video games).

Finally, although this study details the iterative design and development process of MindMax, a more in-depth explanation of the theory behind MindMax, including its applied games components, has been previously published [48]. As per the recommendations of Tondello et al [51], MindMax was also designed to incorporate multiple types of applied games to appeal to a wide variety of users.

#### Interview Protocol

User experience testing interviews followed a semistructured format. We first collected demographic information, specifically gender, age, mobile phone model, and operating system, and how many hours per week participants spent playing video games and watching AFL matches. We then explained to participants that interviews would be conducted using a think-aloud protocol, where participants verbalize their thoughts while completing a series of predetermined tasks. Although there are weaknesses with think-aloud protocols, such as their reliance on participant subjectivity and their inability to capture subconscious cognitive processes, concurrent think-aloud protocols nonetheless have the ability to capture crucial insights at a low cost [52]. The predetermined tasks included procedures such as registering an account, creating an avatar, playing a casual game, and completing psychoeducation modules. The predetermined task list spanned all contemporarily available app features and was hence updated for each subsequent time point.

Participants were allowed to complete the tasks in their preferred order. They were given flexibility as to which modules they chose but were on occasion directed to complete specific modules to ensure an even spread of feedback. All participants completed at least two modules. Although participants were given the opportunity to ask questions during the interview, they were encouraged to complete the tasks unprompted and to the best of their ability. A researcher typed notes of the process.

## Results

### Participant Characteristics

Due to the convenience sampling strategy employed while recruiting for PD workshops (phase 1), participants naturally tended toward video game design students in Brisbane, AFLPA-affiliated individuals in Melbourne, and mental health and technology academics and students in Sydney. In Brisbane and Melbourne, the workshops were divided into 2 groups: aged 16 to 25 years and aged 26 to 35 years. Workshop and participant characteristics are summarized in Table 1 and listed in the chronological order they were conducted.

Table 2 shows the characteristics of the user experience testing interviews and participants (phase 3). One participant (aged 39 years) at the second time point (March 2017) was discovered not to fall within the age range of the target audience, but we made the decision to proceed to gain any relevant insights the participant had to offer toward making the app more broadly accessible. Finally, to eliminate the possibility of bias arising from previously brainstorming this topic at length, user experience interviewees were different across all time points and none had previously attended any MindMax PD workshops.

### Participatory Design Workshop: Descriptive Content Analysis

App likes and dislikes were collected directly during the evaluation phase of PD workshops, where we asked participants to annotate screenshots of multiple existing health and well-being apps/websites. These preferences were coded by an independent knowledge translation team consisting of young people (younger than 25 years) according to the semantic themes content, design, and features/concept. Codes that were observed 3 or more times are presented below.

#### Content

Compared with the other 2 semantic themes, content had the least observations. The most frequently observed preference was that participants disliked activities perceived to be “useless”, “anticlimactic”, “simplistic”, “condescending”, “childish”, or “boring”. Although they were receptive to activities promoting self-reflection, participants disliked vague suggestions (eg, “do something kind for yourself”) and liked having examples of how to do so. Ultimately, participants preferred information that was clear, nonrepetitive, and instructive (not descriptive—“tell me how, not why”). Although participants appreciated explanations for how an activity would benefit them, they preferred this information to be contained within a collapsible content box they could open if desired.

#### Design

Participants overwhelmingly expressed dislikes of excessive blank space and also excessive amounts of text. “Gimmicky” user interfaces (UIs) and graphics received more criticism for looking “childish” and “cheap” than praise for looking “interesting” and having “cool colours”. Furthermore, dark colors such as dark green and brown were criticized for being “depressing” and “ugly”. Instead, participants preferred more conservative UIs with multiple pleasant colors (eg, pastel colors or light to medium blue) paired with simple graphics and names that clearly described the purpose and features of the app. Finally, participants liked how one app listed the number of people that had joined a particular psychoeducational course, as it showed the course appeared to be popular and, therefore, worth trying.

**Table 1:** Participatory design workshop and participant characteristics.

Workshop (n=40), n	Location	Age group (years)	Gender split	Recruitment pool
1 (6)	Melbourne	16-25	3 males, 3 females	AFLPA^a^-affiliated individuals
2 (5)	Melbourne	26-35	3 males, 2 females	AFLPA^a^-affiliated individuals
3 (10)	Sydney	Mixed	4 males, 6 females	Mental health and technology academics and research students
4 (4)	Sydney	Mixed	2 males, 2 females	Mental health and technology academics and research students
5 (8)	Brisbane	16-25	8 males	Video game design and research students and staff
6 (7)	Brisbane	26-35	5 males, 2 females	Video game design and research students and staff

^a^AFLPA: Australian Football League Players’ Association.

**Table 2:** User experience testing interview and participant characteristics.

Time point and participant		Age (years)	Gender	Mobile phone (operating system, OS)	Video game play (hour/week)	AFL^a^ match watching (hour/week)
**December 2016: Web-based alpha prototype (n=4)**						
	P1	29	Female	iPhone 6 (iOS 10.1)	0	1
	P2	33	Female	iPhone 5 (iOS 9)	0.5	1
	P3	26	Male	iPhone 6 (iOS 10.1)	1	1
	P4	29	Female	Samsung S7 Edge (Android; OS unsure)	0	0
**March 2017: native app beta (n=5)**						
	P5	19	Male	iPhone 6 (iOS 10.2.1)	1	2
	P6	24	Male	iPhone 6 (iOS 10.2.1)	20	2
	P7	39	Female	iPhone 6 (iOS 10.2.1)	20	0
	P8	21	Female	iPhone 6 (iOS 10.2.1)	3	0
	P9	20	Male	iPhone 6 (iOS 10.2.1)	30	0
**November 2017: native app 5 months after launch (n=6)**						
	P10	19	Male	Xiaomi RedMe Note 4 (Android 6.0)	5	0
	P11	22	Male	Oneplus 3T (Android 6.0)	5	0
	P12	22	Male	Samsung S5 (Android 6.0.1)	5	0
	P13	22	Male	Samsung J7 Prime (Android 6.0.1)	20	0
	P14	22	Male	Samsung Galaxy S5 (Android 6.0.1)	1 on average; 3 in holidays	0
	P15	20	Male	iPhone 5 (iOS 11.0.3)	24	0

^a^AFL: Australian Football League.

#### Features/Concept

A large number of participants liked the idea of graphs and similar indicators such as goal progress bars, finding it motivational to track their progress. Similarly, a large number of participants also liked the concept of challenges/missions, encouraging them to go beyond their comfort zone. Quizzes attracted both positive and negative feedback, though the former was greater than the latter. Although participants liked having long-term goals and unlockable achievements, their reaction to rewards was ambivalent. Those who liked the concept felt they were “helpful and keep people coming back,” whereas those who did not found there to be “no reward for the player outside of a small number going up.” Participants also liked the skeuomorphic activities in several of the presented apps/websites, where participants could interact with the object on the screen similar to real life (such as scrunching up a piece of paper).

Finally, although competition was seen as “motivating” and “healthy” in the context of a physical health app, participants were ambivalent toward how it, and other types of social sharing options, could be implemented in mental health contexts. Participants felt that any social option that mimicked a major social network would be redundant and that they would not use it. Participants also felt that compared with physical health, mental health was a more private issue that complicated social sharing, both for the sharer and the people they would be sharing their mental health status with. In particular, participants raised the inappropriateness of such features for someone with poor mental health or who was in distress. Instead, participants suggested that social features be used to promote social connection and communication. That is, they should be “supportive rather than competitive.”

#### Other Insights

Furthermore, participants emphasized the importance of being able to customize their app experience, for example, through being able to customize their display image or avatar (if appropriate) or by having their responses to in-app questions influence their app notifications or recommendations. Participants also suggested the incorporation of design elements common to video games, including regular content updates, events, team competitions, and cosmetic digital rewards (eg, avatar hairstyles or clothing). Finally, the issue of mental health stigma was raised, and participants suggested the app have a function for facilitating conversations between men, for example, scheduling real-life activities between friends, where difficult topics could be broached in shoulder-to-shoulder conversations. Participants also specified that the app should adopt an approach of self-improvement, rather than fixing a deficiency.

### User Experience Testing Interviews

#### Summary Across Time Points

Below, we present insights participants expressed during the user experience testing interviews and relevant quotes.

At alpha and beta build, participant feedback comprised identifying software bugs and glitches, criticizing unintuitive UIs and unclear wording, and raising privacy concerns. However, participants also expressed appreciation for the opportunity to see a more personal side of AFL athletes and for the underlying concept of the app:

It’s great! I like the idea and concept of it. Mindfulness, wellbeing. What it’s trying to achieve.

P5

[It was] entertaining, kinda helpful—showed me a lot of values I didn’t know about—so, informative, engaging.

P9

At 5 months after launch, negative participant feedback included identifying software bugs and glitches as well as questioning whether the social component would be used and critiquing its similarity to existing social networks such as Instagram.

However, most participants at this time point found MindMax to meet their user experience standards and to be an overall positive experience that provided some value:

I think overall this app is just for people to try out for curiosity. [...] Sometimes you want to say things to vent, but you can’t really say things to an app. This is like the fries, if the psychologist is a Big Mac.

P13

It’s nice to have prominent masculine role models showing it’s okay to express emotion. Actively saying it’s okay to do so seems like a good thing to do for males in general.

P14

Privacy concerns were raised across all time points. Although participants felt the information they provided MindMax was not particularly sensitive (and some provided false information to MindMax as a further precaution), they were worried that this information would be mistreated (eg, sold to marketers).

#### Content and Delivery

A total of 2 participants were red-green color blind and expressed that the colors used within MindMax were easy to differentiate.

There was a wide range of reactions to the casual tone of the app. Some participants appreciated it:

Good sense of humour. It like makes you feel relaxed.

P5

If it’s too formal, I feel a bit of pressure.

P6

However, on the other hand, some disliked it:

It’s cringey, like those Facebook memes. Makes me take it less seriously.

P14

Participants also asked for more detail and specific, contextualizing examples:

Having an example [...] guidance as to what kind of behaviours are definitive of these values. [...] [Something] more personalised to my chosen value.

P14

Videos were commonly skipped or watched for only the first few seconds. Participants requested the ability to rewind and fast-forward through videos and an indication of what to expect (through subtitles or a transcript). Although all videos were a maximum of 90 seconds long, they were still considered too long:

Once I see a video, I think “this is going to take a while.” Maybe if there was the length of the video on the bottom left or bottom right. If it was like 10 seconds I might watch it.

P11

On 1 occasion, a participant skipped a video that contained key context explaining a later activity in the module, leading to brief confusion.

#### Interactive Activities

MindMax’s psychoeducational modules contained a variety of short interactive activities illustrative of the information in the modules. These activities ranged from uploading selfies and creating “shareables” (eg, a “MaxFive” plan for improving well-being) to share on the community feed, to more in-depth, reflective activities such as guided meditation.

Participant reactions to the social activities were mixed. Although some participants thought they were *different* and *new*, others felt they were inappropriate:

There’s places for selfies and this is not one of them.

P9

(In response to negative feedback to the tone of the activity and privacy concerns, the selfie activity was removed in the launch version of the module.)

Participants who completed the *Mindfulness* and *Emotions* modules tended to skip the guided meditation activity halfway through but were also overall more positive about the activity:

That was pretty cool. [...] I don’t meditate usually so this was a nice experience.

P11

Overall, participants were more positive about activities that required more focus and active participation, particularly in the context of MindMax’s psychoeducational modules being presented as *Training*:

If I didn’t have to physically write the postcard it wouldn’t have resonated so much.

P15

The Mindfulness module fits the concept of “Train” the most. Thinking of a motivational quote and putting it on a picture isn’t training.

P11

#### Avatar

All female participants perceived avatar customization choices to lack feminine options and expressed feelings of alienation:

None of the options look like me, so I’ll make something that’s representative of something else.

P4

This was exacerbated by the default avatar (presented as a base for users to customize) having a mustache. Female participants preferred starting with a blank avatar to this. Male participants did not report feeling alienated by the avatar customization choices.

Although participants preferred the freedom of being able to upload their own profile picture, the majority recognized that uploaded pictures would be difficult to moderate and that avatars provided increased anonymity.

#### Social

Some participants were wary about posting content to the community feed, expressing not only privacy concerns but also more general image management concerns:

[While filling in a shareable]

I guess this would be posted to the main board or something?

Facilitator: Yes.

Ah. So I don’t want to write something too silly.

P15

Overall, participants were negative toward the social component of the app, thinking it was unnecessary and that neither they nor anyone else would use it:

Why care about likes, I’m here to improve mental wellbeing.

P12

Everything’s on Facebook already. I don’t use any other apps other than Facebook to communicate with others.

P15

When asked, most said they would not consciously post anything to the feed (beyond shareables, which were automatically posted through completing modules), though they were more willing to interact with posts on the feed.

Ultimately, participants wanted to use the app with people they already knew or had something in common with:

I just realised you can’t friend people in this, which, I don’t know. With AFL. If I used it a bit longer I would have eventually thought “Why can’t I join a group of just my AFL team rather than everyone?”

P11

[I would like to see] a concept of Circles or Groups [...] I want a way to connect with friends. Like if a few of your friends had similar goals—or putting people with similar goals in a community together.

P5

#### Applied Game Elements

Most participants stated they had never seen games combined with mental health and well-being before and expressed appreciation for the concept. When prompted to spend a “footy” (earned by completing psychoeducational modules and posting in the community feed) to play the casual game Flick Footy, participants at the final time point found the controls intuitive and the experience enjoyable. However, although it was broadly enjoyed, some participants also found it unoriginal and potentially not compelling enough to keep them using MindMax.

One participant found the integration of games and gameful elements within MindMax clumsy and half-hearted:

The app makes it seem the video games section is important, but it’s more than secondary—it’s so far from the general approach of the app that it seems put in in the last minute. Which is fine, but don’t make it seem so important.

P14

The same participant also questioned whether it was appropriate to tie such a simple game so strongly to MindMax’s reward system:

I like video games, and these are very rudimentary, not very interactive, not very engaging [...] if these are the rewards for the activity you’re doing it’s a low reward for something so personal that you have to engage in. Games kind of cheapen the experience.

P14

## Discussion

### Principal Findings

This series of studies aimed to use PD and user testing methods to determine the best way to present MindMax, an AFL-themed app aiming to deliver psychoeducation on mental health and well-being and to create a Web-based community centering on well-being, AFL, and video games. Our results suggest that the concept of combining mental health and well-being with sports and video games was well received by users. Participants gave further insights throughout MindMax’s development period that future mental health and well-being mHealth initiatives can learn from, whether or not they intend to incorporate applied games.

A consistent finding across PD workshops and user experience interviews was that participants did not have strong feelings about what content was presented, but rather how it was presented. Although participants found being presented with too much information at once off-putting, they did not want to be deprived of additional, contextualizing content as a result. Instead, they wanted to be able to control the flow of information, for example, through collapsible content boxes, and to have alternate modes of information, for example, through video subtitles and transcripts. Videos, in particular, were skipped on multiple occasions. This, along with previous research [4], suggests that key information should be presented in a variety of mediums in a way that minimizes repetitiveness.

Participants were sensitive to the formality (and lack thereof) of the various tones adopted by the health and well-being apps presented in PD workshops and in MindMax. Although a formal tone was perceived as intimidating, participants considered an informal tone less pressuring, but some also took the content less seriously as a result. Participants also did not want the tone to be patronizing or paternalistic and yet appreciated specific, direct instructions. Careful writing is needed to achieve this balance. Appropriate levels of formality and prescriptiveness likely vary based on the target audience and must be tested thoroughly with target users. Participants also expected a personalized experience [40], anticipating that MindMax would remember information they entered about themselves (eg, gender or the value they wanted to affirm) and that it would use that information in ways to help the user improve their well-being.

We also observed that participants preferred activities that required active engagement and practiced useful skills, paralleling earlier research on the topic [7]. Activities such as guided meditation and writing a postcard to a loved one, which required comparatively more effort to complete, were better received than activities such as posting a selfie or choosing a quote from a preselected set of quotes. This suggests that our initial intention of reducing cognitive effort and making modules as easy to complete as possible was ineffective and may have undermined the purpose of MindMax. Instead, these findings suggest that user effort should be funneled toward completing activities.

Participants appreciated fun touches to app design (eg, skeuomorphic interactions), but ultimately the majority of participants seemed to prioritize the functional aspects of health apps, such as the ability to track their own health and well-being and information on how to improve their health and well-being. This does not mean there is no room for fun and enjoyment in mental health and well-being apps (multiple successful implementations of gamification in mental health and well-being [30,31] support this). Rather, any implementation of playfulness or supplementary conceptual flavor should be considered and tested carefully.

### Social and Applied Game Elements

Participants overall agreed that social motivations were important to attract and keep them engaged with a mental health and well-being app and that they would prefer the social component to be in relation to their existing social networks. Although they found social comparison and competition (both common gamification features [28]) motivating for improving physical health, they felt that incorporating comparison and competition in a mental health and well-being app could be inappropriate, particularly in cases where the user or someone in their network was in distress. During user experience testing, we observed image management behaviors where participants moderated how they expressed themselves as they were conscious of their potential audience. This was not a desired user behavior and could potentially undermine the improvement of mental health and well-being.

In PD workshops, participants suggested that MindMax and similar apps should complement and enable, rather than emulate, online and offline social connection. Crucially, this social component should be a different experience compared with using a mainstream social networking service. This was difficult to implement in practice, and MindMax ended up failing to follow this recommendation, instead implementing a community feed that user experience interviewees found extremely similar to Instagram. However, future conceptualizations of a social component could draw inspiration from cooperative games and incorporate more gamification elements that appeal to multiple types of users (players), such as social discovery, gifting, and unlockable content [51].

Our user experience interviewees, mostly experienced video game players, also expressed concerns that MindMax’s implementations of applied games lacked depth, which undermined their user experience. Their familiarity with video games may have contributed to their reduced interest in Flick Footy, which was a simple casual game with only 1 aim (score as many goals as possible). In addition to offering a unique, engaging experience, a more sophisticated implementation of cooperative game mechanics could potentially address these concerns and increase MindMax’s appeal to more hardcore video game fans.

### Limitations

The largest limitation of our findings is that during the user experience interview stages, we recruited locally (in Sydney) and therefore found it difficult to recruit AFL fans. However, much of our feedback was not AFL-related and would be useful to anyone designing an app incorporating applied games for mental health and well-being. MindMax’s usage analytics will give an indication of how people interested in AFL perceive and use MindMax and will be the focus of future investigation.

Another limitation is that although the iterative nature of our user testing allowed us to evaluate and improve on subsequent versions of MindMax, in practice, we were limited by financial, technological, and organizational constraints. The time frame of the grant required that development work occur in tandem with the user testing reported in this study. As a result, many features recommended by participants were determined to be unfeasible and descoped. For example, the social component that heavily resembled Instagram was implemented as ultimately there were not enough resources or time to create and implement an alternate concept for the social component. Similarly, although our female participants’ dissatisfaction with the masculine tone of the app was noted, it was not directly actioned given the focus of the project on reaching men in the target age demographic. Finally, given the need to prioritize basic functionality and content inclusion initially, the amount of time available for applied game design and development was reduced, which is reflected in the negative feedback on the games from interviewees with significant video games experience. Although it is also possible that the casual games in MindMax may never have appealed to more experienced video game players, more PD involving more stakeholders than just the research team and potential end users may have led to a smoother development process.

However, participant feedback, especially late-stage feedback, was able to be incorporated later in the project. MindMax underwent continual improvements beyond the time frame covered in this study, introducing new features and events until February 2018. This included new psychoeducational modules, redesigns of UI and existing modules, trophies, team functionality, and a “Flick Footy Max” campaign in December 2017 to promote engagement with the app. In “Flick Footy Max,” MindMax users competed to score the highest in “Flick Footy” to win a PlayStation 4 Pro and a MindMax-themed AFL football. Participant feedback hence continued to influence the development of MindMax beyond what is described in this study.

### Future Applications

MindMax is based in both the AFL and video games subcultures, with the aim of appealing to men who enjoy these subcultures [7]. Although we encountered some difficulties during development, many of which are inevitable on projects with defined time frames and funding windows, we ultimately produced an app containing psychoeducation, applied game elements, and a social component that was considered by user experience interviewees to be satisfactory. We plan to analyze the impact of MindMax via usage analytics and multiple time point survey data assessing users’ levels of well-being and judgments of MindMax’s usability.

Lessons learned from MindMax can be broadly applied to any app intending to help users improve their mental health and well-being, especially those planning to incorporate applied games. They can also be extended to other traditional sporting codes such as cricket, rugby, and soccer, and furthermore, to esports. The increasing collaboration between traditional sports and esports [13] and the growing popularity of esports among younger people [12] may make a broad sports approach including esports suitable to apply to youth mental health. Finally, in the same way that traditional sporting codes such as AFL, rugby union, rugby league, and cricket are now promoting awareness of mental health problems, esports leagues can consider doing the same.

### Conclusions

This study details the PD workshops and user experience testing that was conducted to obtain insights on how best to present MindMax. As an AFLPA initiative funded by Movember, MindMax presents a novel approach to evidence-based mental health and well-being education focusing on Australian men aged 16 to 35 years who enjoy AFL or video games. MindMax incorporates applied games and is couched in the Australian rules football (specifically AFL) subculture. If the implementation of MindMax is successful, there is the potential to generalize its model to other sporting codes such as rugby and cricket and even to partner with esports initiatives.

## Abbreviations

AFL: Australian Football League
AFLPA: Australian Football League Players’ Association
mHealth: mobile health
PD: participatory design
UI: user interface
